# Dyslipidemia and cardiovascular disease risk among the MASHAD study population

**DOI:** 10.1186/s12944-020-01204-y

**Published:** 2020-03-16

**Authors:** Mahshad Hedayatnia, Zahra Asadi, Reza Zare-Feyzabadi, Mahdiyeh Yaghooti-Khorasani, Hamideh Ghazizadeh, Roshanak Ghaffarian-Zirak, Abolfazl Nosrati-Tirkani, Maryam Mohammadi-Bajgiran, Mohadese Rohban, Fatemeh Sadabadi, Hamid-Reza Rahimi, Marzieh Ghalandari, Mohammad-Seddigh Ghaffari, Asa Yousefi, Elnaz Pouresmaeili, Mohammad-Reza Besharatlou, Mohsen Moohebati, Gordon A. Ferns, Habibollah Esmaily, Majid Ghayour-Mobarhan

**Affiliations:** 1 Department of Nutrition, Faculty of Medicine, Mashhad University of Medical Sciences, Mashhad, Iran; 2 Student Research Committee, Mashhad University of Medical Sciences, Mashhad, Iran; 3 Metabolic Syndrome Research Center, School of Medicine, Mashhad University of Medical Sciences, Mashhad, Iran; 4 International UNESCO Center for Health-Related Basic Sciences and Human Nutrition, Department of Nutrition, School of Medicine, Mashhad University of Medical Sciences, Mashhad, Iran; 5 Faculty of Medicine, Mashhad University of Medical Sciences, Mashhad, Iran; 6 Cardiovascular Research Center, School of Medicine, Mashhad University of Medical Sciences, Mashhad, Iran; 7 Division of Medical Education, Brighton & Sussex Medical School, Falmer, Brighton, Sussex UK; 8 Social Determinants of Health Research Center, Mashhad University of Medical Sciences, Mashhad, Iran

**Keywords:** Dyslipidemia, Cardiovascular disease, Stable angina, Unstable angina, Myocardial infarction, Total cholesterol

## Abstract

**Introduction:**

Dyslipidemia may be defined as increased levels of serum total cholesterol (TC), low-density lipoprotein cholesterol (LDL-C), triglycerides (TG), or a decreased serum high-density lipoprotein cholesterol (HDL-C) concentration. Dyslipidemia is an established risk factor for cardiovascular disease (CVD). We aimed to investigate the association of dyslipidemia and CVD events among a population sample from Mashhad, in northeastern Iran.

**Material and methods:**

This prospective cohort study comprised a population of 8698 men and women aged 35–65 years who were recruited from the Mashhad Stroke and Heart Atherosclerotic Disorder (MASHAD) study. Socioeconomic and demographic status, anthropometric parameters, laboratory evaluations, lifestyle factors, and medical history were gathered through a comprehensive questionnaire and laboratory and clinical assessment for all participants. Cox regression model and 95% confidence interval (CI) were used to evaluate the association of dyslipidemia and its components with CVD incidence.

**Results:**

After 6 years of follow-up, 233 cases of CVD (including 119 cases of unstable angina [US], 74 cases of stable angina [SA], and 40 cases of myocardial infarction [MI]) were identified in the study population. Unadjusted baseline serum LDL-C, TC, and TG levels were positively associated with the risk of total CVD events among the entire population (HR: 1.54, 95% CI: 1.19–2; *P*-value< 0.01; HR: 1.53; 95% CI: 1.18–1.98; *P* < 0.01; HR: 1.57; 95% CI: 1.27–2.03; P < 0.01, respectively). However, after adjusting for confounding factors (age, body mass index [BMI], family history of CVD, smoking status [non-smoker, ex-smoker and current smoker], lipid lowering drug treatment, anti-hypertensive drug treatment, hypertension, healthy eating index [HEI], total energy intake, and presence of diabetes mellitus), a significant direct association only remained between TC and MI risk in men (HR: 2.71; 95%CI: 1.12–6.57; *P*-value< 0.05).

**Conclusion:**

In the present study, TC baseline level was significantly associated with the risk of MI among men.

## Introduction

Dyslipidemia is characterized by an elevation of serum total cholesterol (TC), low-density lipoprotein cholesterol (LDL-C), or triglycerides (TG) and reduced serum high-density lipoprotein cholesterol (HDL-C) concentration [1–3] and is these are routinely assessed for the purpose of assessing cardiovascular risk. The prevalence of dyslipidemia varies geographically; although, it has been estimated that more than 50% of the adult population has dyslipidemia worldwide [4–6]. The prevalence of hypercholesterolemia, hypertriglyceridemia, high levels of LDL-C, and low levels of HDL-C are reported to be 41.6, 46.0, 35.5, and 43.9%, respectively in both sexes in the Iranian population [7]. Darroudi et al. have recently reported the prevalence of dyslipidemia among a subsample of Iranian adults to be 83 and 87% in the total population and CVD patients, respectively [8].

CVD is a chronic non-communicable disease and one of the most important causes of death and disability [9]. The prevalence of CVD events is increasing globally [9, 10]. It is the leading cause of mortality in Iran, accounting for 50% of total mortality and 79% of deaths due to chronic diseases [9]. Atherosclerosis is the major underlying cause of CVD [11]; the World Health Organization (WHO) definition of CVD includes: coronary heart disease, cerebrovascular disease, rheumatic heart disease, myocardial infarction (MI), stable angina (SA), unstable angina (UA), and other conditions [11]. Public health organizations globally have focused on reducing modifiable CVD risk factors to control the rising prevalence of CVD and its risk factors; such as hypertension (HTN), unhealthy diet, obesity and dyslipidemia [3, 10, 12–14]. A high-fat and high-calorie diet can cause dyslipidemia and thereafter endothelial dysfunction [15]. Serum TG, TC, LDL-C, HDL-C, TC/HDL-C, and LDL-C/HDL-C ratios are independent predictors of CVD risk. Currently, the principal objective in the management of dyslipidemia is to reduce serum LDL-C levels [16].

Components of the circulating lipid profile, but particularly modified LDL-C, may be deposited within the tunica intima of the artery wall, and are involved in the subsequent atherogenic process [17–19]. The benefits of reducing plasma LDL-C concentrations on CVD risk are particularly evident in subjects with familial hypercholesterolemia [20]. Although the association of high TG levels with the occurrence of cardiovascular disease (CVD), especially atherosclerotic CVD, has been well documented in large cohort studies [21], its role as an independent CVD risk factor remains controversial [20]. The reason for this is that an elevated TG concentration is associated with higher concentrations of the atherogenic small dense LDL particles and lower HDL-C concentrations [20].

Whilst it has been proposed that HDL-C is protective against CVD, partially related to its role in reverse cholesterol transport [2, 22], some studies have reported that high or normal levels of HDL-C are not protective against CVD events [23]. A single serum HDL-C level reflects the HDL-C pool rather than its functionality [23]. Modified forms of the various protein components of HDL, perhaps generated by oxidative stress, may reduce the ability of HDL to take part in reverse transport [23].

Whilst several studies have demonstrated the association of serum lipid levels and the occurrence of CVD in western population samples [24–27], there, are relatively few in Asian populations. We aimed to define the association between all components of dyslipidemia and the incidence of CVD events among a population sample of adults from north-eastern Iran.

## Materials and methods

### Study population

Participants were recruited as part of the Mashhad stroke and heart atherosclerotic disorder (MASHAD) cohort study. The MASHAD study is a prospective cohort study, comprising 9704 men and women aged 35–65 years from the city of Mashhad, in the country of Iran. This study was initiated in 2010 and was planned to evaluate various CVD risk factors (code no: 85134). The incidence of CVD was assessed among participants at several follow-up time-points (2011, 2014 and 2016). Individuals who were lost to attendance at the different stages of follow-up or those with missing data regarding lipid profile components (including LDL-C, TC, TG, and HDL-C) were excluded from the study. Finally, 8698 subjects including 235 individuals with CVD, and 8465 individuals without overt CVD were included in the data analysis. The Human Research Ethics Committee of Mashhad University of Medical Sciences (MUMS) reviewed and approved the study. All participants provided written informed consent.

### Diagnosis of cardiovascular diseases

The occurrence of CVD among participants at follow-up was ascertained by taking a detailed medical history, followed by a physical examination by a specialist Cardiologist. Electrocardiograms were checked by the cardiologist for the evidence of alterations in P, QRS, T and especially Q wave by using the Minnesota Code [28, 29]. If the cardiologist suspected a diagnosis of CVD, further examinations were undertaken that included; echocardiography, stress echocardiography, radioisotope, angiography, Computed Tomography (CT) angiography, and Exercise Tolerance Test (ETT). The definitive diagnosis was based on the consensus agreement of a panel of experts among 235 subjects including 120 subjects with unstable angina, 75 subjects with stable angina, and 40 subjects with myocardial infarction. Finally, we excluded 2 CVD patients due to missing data regarding lipid profile (including 1 subject with unstable angina and 1 subject with stable angina).

### Anthropometric assessments

Height, weight, body mass index (BMI), waist circumference (WC), hip circumference (HC), waist-to-hip ratio (WHR), and mid-upper arm circumference (MAC) were measured according to the standardized protocols [30] in all participants. Height (cm), waist circumference (cm), hip circumference (cm), and mid-upper arm circumference (cm) were measured to the nearest millimeter using a tape measure. Weight (kg) was measured to the nearest 0.1 kg using electronic scales. The BMI was calculated by dividing weight (kg) to height squared (m^2^) [30]. Interpretation of the mean BMI was admitted by the standardized protocols [31, 32]. Waist-to-hip ratio (WHR) was calculated by dividing waist circumference to hip circumference [30].

### Laboratory evaluation

Blood samples of all participants were collected after a 14 h overnight fast. Serum TG, low-density lipoprotein cholesterol (LDL-C), high-density lipoprotein cholesterol (HDL-C), and total cholesterol (TC), were estimated using enzymatic methods on an automated analyzer; fasting blood glucose (FBG) was also measured by enzymatic methods on an autoanalyser. Dyslipidemia was defined as TC ≥ 200 mg/dl, or TG ≥150 mg/dl, or LDL-C ≥ 130 mg/dl, or HDL-C < 40 mg/dl (for men) and HDL-C < 50 (for women) [5, 33, 34]. Diabetes was defined as FBG ≥126 mg/dl [5, 35].

### Blood pressure assessment

Systolic blood pressure (SBP) and diastolic blood pressure (DBP) were measured using calibrated mercury sphygmomanometers. HTN in the cases who had high blood pressure was defined as systolic blood pressure ≥ 140 mmHg or diastolic blood pressure ≥ 90 mmHg [36].

### Assessment of physical activity level (PAL)

The questionnaire used for assessing physical activity was based on the James and Schofield human energy requirements equations and was completed by all subjects. Questions were divided into three parts that included activities in work time, non-work time, and in bed [37–39].

### Dietary intake assessment and healthy eating index calculation

The dietary intake and total energy consumption (kcal/day) of all participants were determined by using a validated 65-items food frequency questionnaire (FFQ), which was completed by a trained nutritionist for the entire population of the study at baseline. Each item of the FFQ included the portion size and five frequency groups (consumption per day, per week, per month, rarely, and never). The consumption of each food item (gram per day) was obtained by multiplying the portion size and reported frequency of intake of that food item [40]. The intra-class correlation coefficient (ICC) which represents a good validity of most food items was calculated by the agreement of the FFQ and 24-h recall. A Pearson’s correlation coefficient indicated good reproducibility of the FFQ by a correlation between FFQ1 and FFQ2 that were completed with a definite interval for a sample of the participants [41]. To assess diet quality, the Healthy-Eating-Index2010 (HEI-2010) was used as a reliable tool [42]. The scores for each component ranged from zero to 10 points. The protein, foods, vegetables, and fruits have a maximum score of 5 points. Empty calorie components’ scores are assigned up to 20 points. The summation of the component scores can reach up to 100 points. The mean total healthy eating index-2010 scores were used in the results section. The HEI index includes multiple component systems scoring to characterize the quality of diet [43]. The latest version of HEI is called HEI-2015. It contains 13 components that reflect the different food groups and critical recommendations in the 2015–2020 Dietary Guidelines for Americans, including total fruit, whole fruit, vegetables, greens and beans, whole grains, dairy, total protein foods, seafood and plant proteins, and fatty acids (FAs). The second group assesses dietary components that should be consumed in moderation, namely, refined grains, sodium, and empty calories. Empty calories are defined as energy consumption from added sugars, alcohol, and solid fats [44]. In this study, we used a version of HEI 2010 [45].

### Assessment of other variables

Demographic and socioeconomic characteristics (e.g. age, sex, marriage status and education), lifestyle data including smoking (current smoker, ex-smoker and non-smoker), drug history (DH) (include lipid lowering drug and anti-hypertensive drug), and family history (FH) of CVD for all participants were collected by health care professionals and a nurse interview.

### Statistical analysis

All statistical analyses were performed using SPSS version 20 (SPSS, Chicago, IL). The chi-square, analysis of variance (ANOVA), and Kruskal-Wallis tests were used to report distribution differences for qualitative and quantitative (normal and non-normal) data, respectively among defined groups of the study population. Hazard ratios (HRs) and 95% confidence intervals (CIs) were calculated using cox regression to obtain the association of dyslipidemia and CVD risk. The calculated HRs were adjusted for potential confounders including age, total energy intake, HEI, body mass index (BMI), family history of CVD, smoking status (non-smoker, ex-smoker and current smoker), lipid lowering drugs, anti-hypertensive drug treatment, presence of diabetes mellitus, and hypertension. A *p*-value of less than 0.05 was considered as significant.

## Results

After excluding 1006 persons who were lost to follow-up, or those with missing data, we included 8698 participants of the MASHAD cohort study in the final analysis. Of these, 233 subjects (including 119 cases of unstable angina, 74 cases of stable angina, and 40 cases of MI) had suffered a CVD event and 8465 subjects were considered to be healthy controls, and were included in the final data analysis.

Participants were compared based on the occurrence of CVD (Table 1). The percentage of males within the case and control groups were 47 and 41%, respectively. Subjects who had CVD events were older than the control group (*p*-value < 0.001). We also found that CVD patients tended to be less well educated, more had a positive smoking history (ex-smoker and current smokers) compared to healthy controls (*p*-value < 0.001). Weight, BMI, waist circumference (WC), and waist-to-hip ratio (WHR) were higher in the CVD patients (*p*-values < 0.01) while, healthy participants had higher levels of physical activity level (PAL) (*p*-value = 0.001). The levels of serum TC, TG, LDL-C and FBG were all higher in CVD patients (*p*-values < 0.01), whereas serum HDL-C concentrations were lower (*p*-value < 0.001). Systolic and diastolic blood pressures were higher in CVD patients. The prevalence of dyslipidemia, hypertension, diabetes, and family history of CVD were greater in CVD patients (*p*-value < 0.001). Lipid lowering drug and anti-hypertensive drug treatment were higher in the CVD patients than for the control group (*p*-value < 0.001).

**Table 1:** Baseline characteristic of participants who did or did not develop cardiovascular disease at follow-up

Variable	CVD		*p*-value
	No (*n* = 8465)	Yes (*n* = 233)	
Male, % (N)	41 (3473)	47 (109)	0.069
Age, year	47.85 ± 8.12	54.31 ± 6.98	< 0.001
Marriage status, % (N)			
Married	93.80 (7941)	92.20 (215)	0.326
Single/divorced/widow	6.20 (523)	7.80 (18)	
Education level, % (N)			
Low (trade school)	52.40 (4432)	65.90 (154)	
Moderate (high school)	35.90 (3033)	25 (58)	< 0.001
High (university)	11.70 (989)	9.10 (21)	
Smoking status, % (N)			
Non smoker	69.60 (5883)	62.10 (145)	
Ex – smoker	9.70 (823)	16.80 (39)	0.001
Current smoker	20.70 (1752)	21.10 (49)	
PAL	1.59 ± 0.28	1.53 ± 0.28	0.001
Weight (kg)	71.85 ± 12.84	74.15 ± 12.49	0.007
Height (cm)	160.81 ± 9.14	160.27 ± 9.17	0.374
BMI (kg/ m^2^)	27.83 ± 4.70	28.92 ± 4.54	< 0.001
WC (cm)	95.10 ± 12.01	99.05 ± 10.59	< 0.001
HC (cm)	103.65 ± 9.26	104.29 ± 9.41	0.298
WHR	0.92 ± 0.08	0.95 ± 0.07	< 0.001
MAC (cm)	30.58 ± 3.94	30.62 ± 3.62	0.865
Dyslipidemia	66 (5590)	78.90 (183)	< 0.001
Cholesterol (mg/dl)	190.56 ± 38.56	199.83 ± 42.16	< 0.001
TG (mg/dl)	120 (84, 171)	139 (101.25, 215.50)	< 0.001
LDL-C (mg/dl)	115.89 ± 34.96	122.35 ± 35.59	0.006
HDL-C (mg/dl)	42.82 ± 9.87	40.21 ± 9.05	< 0.001
FBG (mg/dl)	91.45 ± 36.92	119.45 ± 63.10	< 0.001
SBP (mmHg)	121.26 ± 18.22	134 ± 20.88	< 0.001
DBP (mmHg)	79.01 ± 11.74	83.77 ± 10.69	< 0.001
HTN, %	22.60 (1907)	44.80 (104)	< 0.001
DM, %	8.30 (704)	28 (65)	< 0.001
Lipid lowering drug, % (N)	2.40 (203)	12.90 (30)	< 0.001
Anti-hypertensive drug, % (N)	4.10 (347)	16.40 (38)	< 0.001
Family history of CVD, % (N)	34.90 (2929)	44.30 (101)	0.003
HEI	55.27 ± 8.56	55.71 ± 8.27	0.433
Total energy intake (kcal/day)	2022.46 ± 679.19	1945.66 ± 708.74	0.148

*Abbreviations*: *PAL* physical activity level, *BMI* body mass index, *WC* waist circumference, *HC* hip circumference, *WHR* waist-to-hip ratio, *MAC* mid- upper arm circumference, *SBP* systolic blood pressure, *DBP* diastolic blood pressure, *FBG* fasting blood glucose, *TG* triglyceride, *LDL-C* low density lipoprotein cholesterol, *HDL-C* high density lipoprotein cholesterol, and *HEI* healthy eating index

Data are presented as means ± standard deviations and median (interquartile range) for continuous variables and as percentages for categorical variables. Independent sample t-test and Mann-Whitney U test were used where appropriate

Hypertension was defined as systolic blood pressure ≥ 140 mmHg, diastolic blood pressure ≥ 90 mmHg

Diabetes was defined as fasting blood glucose ≥126 /mg/dl

Dyslipidemia was defined as total cholesterol ≥200, or triglycerides ≥150, or low-density lipoprotein cholesterol (LDL-C) ≥130, or high-density lipoprotein cholesterol (HDL-C) < 40 (for men) and HDL-C < 50 (for women)

There are some missing data regarding marriage status (no CVD: 2), education level (no CVD: 12), smoking status (no CVD: 8), HTN (no CVD: 29) and family history of CVD (no CVD: 67, CVD: 4)

In Table 2, we compare all participants with or without dyslipidemia; 7424 participants had dyslipidemia and 1273 did not. Participants with dyslipidemia were older, heavier, shorter, tended not to be married, had higher mean BMI, WC, hip circumference (HC), and WHR (*p*-value < 0.05). PAL and mid-upper arm circumference (MAC) were higher in participants without dyslipidemia (*p*-value < 0.001). Unsurprisingly mean serum TC, TG, and LDL-C, and FBG were higher in participants with dyslipidemia; and serum HDL-C was lower than in participants without dyslipidemia (*p*-value < 0.001). Systolic and diastolic blood pressures, the prevalence of hypertension, diabetes, and family history of CVD were higher in the participants with dyslipidemia (*p*-value < 0.001). `A higher percentage of subjects in the non dyslipidemia group were taking lipid lowering drugs, and a higher percentage of those with dyslipidemia consumed anti-hypertensive drugs (*p*-value < 0.001). The HEI was higher among individuals with dyslipidemia, while total energy intake was lower among this group compared to those without dyslipidemia (*p*-value < 0.001).

**Table 2:** Baseline characteristics of participants based on the presence or absence of dyslipidemia

Variables	Dyslipidemia	Dyslipidemia	*P*-value
	Yes (*n* = 7424)	No (*n* = 1273)	
Age, year	48.18 ± 8.10	47.10 ± 8.42	< 0.001
Marriage status, %(N)			
Married	93.50 (6941)	95.30 (1213)	0.016
Single/divorced/widow	6.50 (481)	4.70 (60)	
Education level, % (N)			
Low (trade school)	52.70 (3909)	53.10 (675)	
Moderate (high school)	35.60 (2641)	35.40 (450)	0.976
High (university)	11.60 (863)	11.60 (147)	
Smoking status, % (N)			
Non smoker	69.60 (5162)	67.90 (864)	
Ex – smoker	9.80 (724)	10.80 (138)	0.384
Current smoker	20.60 (1531)	21.20 (270)	
PAL	1.58 ± 0.28	1.65 ± 0.31	< 0.001
Weight (kg)	72.61 ± 12.77	67.86 ± 12.49	< 0.001
Height (cm)	160.52 ± 9.10	162.42 ± 9.25	< 0.001
BMI (kg/ m^2^)	28.21 ± 4.61	25.79 ± 4.69	< 0.001
WC (cm)	95.94 ± 11.84	90.91 ± 11.95	< 0.001
HC (cm)	104.10 ± 9.22	101.13 ± 9.12	< 0.001
WHR	0.92 ± 0.08	0.90 ± 0.08	< 0.001
MAC (cm)	30.07 ± 3.73	30.07 ± 4.91	< 0.001
Cholesterol (mg/dl)	194.51 ± 39.96	169.20 ± 19.20	< 0.001
TG (mg/dl)	152.52 ± 95.62	83.14 ± 28.28	< 0.001
LDL-C (mg/dl)	119.45 ± 35.78	96.34 ± 21.07	< 0.001
HDL-C (mg/dl)	41.17 ± 9.27	51.92 ± 8.05	< 0.001
FBG (mg/dl)	93.43 ± 39.73	85 ± 25.69	< 0.001
SBP (mmHg)	121.99 ± 18.53	119.29 ± 17.52	< 0.001
DBP (mmHg)	79.38 ± 11.90	77.73 ± 10.65	< 0.001
HTN, % (N)	23.90 (1767)	19.20 (243)	< 0.001
DM, % (N)	9.80 (724)	3.50 (45)	< 0.001
Lipid lowering drug, % (N)	0.90 (11)	3 (222)	< 0.001
Anti-hypertensive drug, % (N)	4.80 (355)	2.40 (30)	< 0.001
Family history of CVD, % (N)	36.10 (2655)	29.70 (375)	< 0.001
HEI	55.44 ± 8.54	54.34 ± 8.56	< 0.001
Total energy intake (kcal/day)	2002.03 ± 654.9	2126.49 ± 806.62	< 0.001

*Abbreviations*: *PAL* physical activity level, *BMI* body mass index, *WC* waist circumference, *HC* hip circumference, *WHR* waist-to-hip ratio, *MAC* mid- upper arm circumference, *SBP* systolic blood pressure, *DBP* diastolic blood pressure, *FBG* fasting blood glucose, *TG* triglyceride, *LDL-C* low density lipoprotein cholesterol, *HDL-C* high density lipoprotein cholesterol, and *HEI* healthy eating index

Data are presented as means ± standard deviations for continuous variables and as percentages for categorical variables

Hypertension was defined as systolic blood pressure ≥ 140 mmHg, diastolic blood pressure ≥ 90 mmHg

Diabetes was defined as fasting blood glucose ≥126 /mg/dl

Dyslipidemia was defined as total cholesterol ≥200, or triglycerides ≥150, or low-density lipoprotein cholesterol (LDL-C) ≥130, or high-density lipoprotein cholesterol (HDL-C) < 40 (for men) and HDL-C < 50 (for women)

There are some missing data regarding marriage status (dyslipidemia patients: 2) education level (dyslipidemia patients: 17, healthy controls: 9), smoking status (dyslipidemia patients: 7, healthy controls: 1), HTN (dyslipidemia patients: 24, healthy controls: 5) and family history of CVD (dyslipidemia patients: 62, healthy controls: 9)

In Table 3, we compare the 3582 men to 5116 women based on having dyslipidemia and its components. We found that the prevalence of dyslipidemia and its components were higher in women, except for serum TG concentration which was higher among men (*p*-value < 0.001).

**Table 3:** The prevalence of dyslipidemia and its components among the study population

	Dyslipidemia	High LDL-C	High TC	High TG	Low HDL-C
Total population (*n* = 8698)	85.40 (7424)	32.30 (2811)	38 (3309)	33.90 (2947)	65.80 (57.19)
Men (*n* = 3582)	80.30 (2877)	29.80 (1067)	34.10 (1223)	36.90 (1321)	55.60 (1990)
Women (*n* = 5116)	88.90 (4547)***	34.10 (1744)***	40.80 (2086)***	31.80 (1626)***	72.90 (3729)***

*Abbreviations*: *LDL-C* low density lipoprotein cholesterol, *TC* total cholesterol, *TG* triglyceride, *HDL-C* high density lipoprotein cholesterol

*** From comparing women with men (*P* < 0.001)

In Table 4, we have evaluated the association between dyslipidemia and its components with the occurrence of CVD (myocardial infarction [MI], stable angina [SA], and unstable angina [UA]). In the total population, according to crude HR, the risk of total CVD was associated with dyslipidemia and high levels of LDL-C, TC, and TG (*p*-value < 0.05). The risk of MI was associated with higher mean levels of LDL-C and TC (*p*-value < 0.05), whilst the risk of SA was associated with high levels of TC (*p*-value < 0.05); though this was no longer the case after adjusting for several confounding factors.

**Table 4:** Hazard ratios (95% CIs) for cardiovascular disease events associated with dyslipidemia and its components

	Dyslipidemia	High LDL-C	High TC	High TG	Low HDL-C
Total population					
Total CVD					
Crude HR (95%CI)	1.81 (1.15–2.87)*	1.54 (1.19–2)**	1.53 (1.18–1.98)**	1.57 (1.21–2.03)**	1.15 (0.87–1.52)
Multivariable HR (95%CI)*	1.14 (0.68–1.89)	1.16 (0.85–1.59)	0.93 (0.68–1.26)	1.13 (0.82–1.55)	0.96 (0.69–1.33)
MI					
Crude HR (95%CI)	2.12 (0.65–6.89)	1.91 (1.03–3.55)*	2 (1.07–3.73)*	1.77 (0.95–3.30)	1.08 (0.56–2.10)
Multivariable HR (95%CI)*	1.90 (0.44–8.07)	1.65 (0.83–3.30)	1.46 (0.72–2.95)	1.52 (0.75–3.09)	0.80 (0.38–1.66)
SA					
Crude HR (95%CI)	1.24 (0.62–2.49)	1.52 (0.96–2.41)	1.73 (1.10–2.73)*	1.50 (0.95–2.37)	1.23 (0.75–2.02)
Multivariable HR (95%CI)*	0.90 (0.40–2.04)	1.10 (0.61–1.97)	1.02 (0.57–1.81)	1.14 (0.63–2.05)	1 (0.55–1.83)
UA					
Crude HR (95%CI)	2.37 (1.15–4.85)	1.43 (0.99–2.06)	1.31 (0.92–1.89)	1.58 (1.10–2.26)	1.14 (0.77–1.68)
Multivariable HR (95%CI)*	1.16 (0.55–2.43)	1.03 (0.66–1.61)	0.73 (0.47–1.14)	1.01 (0.65–1.57)	1.01 (0.63–1.62)
Men					
Total CVD					
Crude HR (95%CI)	1.43 (0.84–2.43)	1.37 (0.93–2.03)	1.36 (0.93–1.99)	1.40 (0.96–2.05)	1.01 (0.69–1.48)
Multivariable HR (95%CI)*	0.95 (0.53–1.71)	1.13 (0.71–1.81)	1 (0.63–1.59)	1.49 (0.94–2.35)	0.90 (0.57–1.41)
MI					
Crude HR (95%CI)	1.81 (0.54–6.03)	1.86 (0.84–4.10)	2.46 (1.12–5.43)*	2.19 (0.99–4.82)	1.02 (0.46–2.24)
Multivariable HR (95%CI)*	1.95 (0.45–8.51)	2.22 (0.94–5.26)	2.71 (1.12–6.57)*	2.35 (0.94–5.86)	0.85 (0.36–2.04)
SA					
Crude HR (95%CI)	0.86 (0.35–2.12)	1 (0.44–2.28)	1.14 (0.52–2.49)	1.38 (0.64–2.94)	1 (0.47–2.13)
Multivariable HR (95%CI)*	0.58 (0.20–1.66)	0.66 (0.22–2.03)	0.62 (0.22–1.75)	1.48 (0.56–3.91)	0.83 (0.33–2.11)
UA					
Crude HR (95%CI)	1.75 (080–3.87)	1.38 (0.81–2.37)	1.13 (0.66–1.93)	1.16 (0.68–1.97)	1.02 (0.61–1.72)
Multivariable HR (95%CI)*	0.91 (0.40–2.11)	1.01 (0.51–1.98)	0.71 (0.36–1.39)	1.18 (0.62–2.24)	0.95 (0.50–1.81)
Women					
Total CVD					
Crude HR (95%CI)	3.71 (1.37–10.04)*	1.74 (1.22–2.48)**	1.75 (1.23–2.50)**	1.70 (1.19–2.42)**	1.52 (0.97–2.38)
Multivariable HR (95%CI)*	1.95 (0.61–6.24)	1.19 (0.77–1.82)	0.83 (0.53–1.28)	0.80 (0.51–1.25)	1.12 (0.67–1.89)
MI					
Crude HR (95%CI)	_¤	2.23 (0.81–6.14)	1.67 (0.60–4.60)	1.08 (0.37–3.16)	2.42 (0.55–10.74)
Multivariable HR (95%CI)*	_¤	1.31 (0.40–4.26)	0.72 (0.21–2.41)	0.67 (0.21–2.20)	1.14 (0.24–5.49)
SA					
Crude HR (95%CI)	1.84 (0.57–5.93)	1.87 (1.06–.31)*	2.16 (1.20–3.86)*	1.61 (0.90–2.87)	1.38 (0.68–2.77)
Multivariable HR (95%CI)*	1.38 (0.32–5.92)	1.30 (0.64–2.67)	1.18 (0.57–2.46)	0.92 (0.43–1.97)	1.08 (0.47–2.45)
UA					
Crude HR (95%CI)	7.55 (1.05–54.46)*	1.51 (0.91–2.49)	1.56 (0.95–2.57)	2.03 (1.23–3.4)**	1.52 (0.81–2.86)
Multivariable HR (95%CI)*	2.75 (0.37–20.22)	1.06 (0.57–1.94)	0.66 (0.36–1.21)	0.77 (0.41–1.43)	1.20 (0.57–2.55)

*Abbreviations*: *CVD* cardiovascular disease, *MI* myocardial infarction, *SA* stable angina, *UA* unstable angina, *HR* hazard ratio, *CI* confidence interval

Dyslipidemia was defined as total cholesterol ≥200, or triglycerides ≥150, or low-density lipoprotein cholesterol (LDL-C) ≥130, or high-density lipoprotein cholesterol (HDL-C) < 40 (for men) and HDL-C < 50 (for women)

*Multivariable HRs were adjusted for age, body mass index (BMI), family history of cardiovascular disease, smoking status (non-smoker, ex-smoker and current smoker), lipid lowering drug, anti-hypertensive drug, diabetes mellitus, hypertension, HEI, and total energy intake

¤ There was no MI event in this category

* *P*-value< 0.05, ** *P*-value< 0.01, *** *P*-value< 0.001

In men, according to crude HR and multivariable HR, a high serum TC was associated with an increased risk of MI (*p*-value < 0.05). In women, according to crude HR dyslipidemia, high levels of LDL-C, TC, and TG were positively associated with total CVD events (*p*-value < 0.05). The risk of SA was associated with high TC, while dyslipidemia and high TG concentrations were associated with an increased risk of UA (*p*-values < 0.05). However after adjusting for potential confounding factors, the significance of these associations did not remain.

## Discussion

Our study aimed to investigate the association of dyslipidemia and its components with CVD incidence after 6 years of follow-up. At this timepoint, 2.68% (*n* = 233) had developed CVD, and dyslipidemia was highly prevalent in the total population; estimated to be 85.35% (*n* = 7424). However, our findings were different before and after multivariable adjustment. According to the crude HR, serum LDL-C, TC, and TG levels were positively associated with total CVD risk among the total population and women. However, after adjustment for confounding factors, including diet, these associations were no longer significant, apart for a high serum TC that significantly increased the risk of MI in men.

Dyslipidemia and lipid oxidation are thought to be important determinants of atherosclerosis that leads to CVD [11]. We aimed to study the relationship between dyslipidemia and CVD in an Iranian adult cohort to find the relationship between dyslipidemia and its components with an incidence of CVD, as there have been inconsistent reports concerning this relationship globally.

There was a significant association between LDL-C and CVD risk in the study population using unadjusted data analysis. LDL-C is considered to be a major risk factor in the incidence of revascularisations, ischaemic strokes, atherothrombotic process and cardiovascular death [46], and cardiovascular guidelines in the US, and also European consider LDL-C as an important modifiable risk factor [47, 48]. This is supported by numerous trials including two recent clinical outcome trials of proprotein convertase subtilisin/kexin 9 (PCSK9) inhibitors that increase the expression of the LDL-C receptor on hepatocytes as well as LDL-C clearance by the liver [49]. These studies have shown that the risk of CVD was reduced substantially in patients with high-risk of atherosclerotic cardiovascular disease (ASCVD) on statin therapy [50, 51].

Wallace et al. and Wilson et al. have demonstrated a direct relationship between serum LDL-C and CVD incidence [52, 53]. It has also been shown that an increased level of TC (hypercholesterolemia), particularly LDL-C promotes the atherosclerosis process, leading to the deposition of cholesterol and fatty acids in the artery wall, whilst HDL-C is usually considered to be protective and returns cholesterol to the liver [2, 3, 19, 27].

After adjustment for confounding factors, high LDL-C levels were not found to be related to the risk of heart failure among the Copenhagen General Population Study and the Copenhagen City Heart Study [54]. Varbo A et al. reported that although remnant cholesterol and LDL-C would cause CVD events to a similar degree, remnant cholesterol causes more severe form of atherosclerosis compared to LDL-C [54]. In the current study high LDL-C level were associated with an increased risk of total CVD before adjusting. After adjusting for all possible confounding factors, these associations were no longer significant. Inconsistent results regarding the relationship between CVD risk and dyslipidemia might be related to the inclusion of different confounding factors in the data analysis. For example, Cromwell et al. adjusted their data only for age, gender, SBP, smoking, and lipid-lowering drugs [55]; and Berg et al. adjusted for age, gender, BMI, smoking, alcohol, diabetes mellitus and lipid-lowering drugs [56].

Previous studies have demonstrated a positive relationship between both a higher LDL-C and lower HDL-C with an increased risk of CVD [52, 53, 57]. These studies had longer follow-up duration and also considered some of the predisposing genetic factors. These inconsistent results may be attributed to the differences in the study population that include: sample size, ethnicity, and environmental factors. Niroumand and coworkers in Iran [3] assessed the atherogenic Index of plasma (AIP) according to the formula, log (TG/HDL-C), in a cross-sectional study [3]. Elevated levels of TG were found to increase the risk of CVD among men more than women, though the role of TG in the pathogenesis of CVD and atheroma formation is still not clear. Nevertheless, it may be related to the cholesterol content of triglyceride-rich lipoproteins rather than the role of TG particles [58, 59]. However, we found no significant association between TG and CVD events in the present study.

We found no significant relationship between serum HDL-C on the incidence of CVD. Whilst, Freitas et al. have reported that low HDL-C is a risk factor for the development of CVD in the elderly [60], a systematic review and meta-analysis reported that increased HDL-C levels was not associated with a reduced risk CVD or CVD mortality [61]. It is possible that VLDL-C and IDL-C are important CVD risk factors in our population; that is non-HDL cholesterol may be associated with an increased the risk of CVD events in our population sample. Lawler et al. showed that participants who consumed statins had lower risk of CVDs by their effects on reducing VLDL-C to a greater extent than LDL-C concentrations [62]. Some studies show that decreasing LDL-C levels, and increasing the size of HDL-C particles, or serum HDL levels do not reduce risk of the CAD [63, 64]. Studies of HDL-C function have also aimed to assess the role of HDL-C in the incidence of CVD events [65]. Recent studies have shown that differences in HDL-C function may be associated with altered cholesterol efflux from cells and may also be independent markers of CVD risk [66, 67]. However, high levels of serum lipoproteins are important risk factors involved in CVD risk calcific aortic valve stenosis, and stroke [68, 69].

We were not able to investigate the association between serum apolipoprotein concentrations on the risk of CVD, which reported previously; Walldius et al. showed that an enhanced level of apolipoprotein B and a reduced level of apolipoprotein A1 are strong predictors of CVD risk [70]. LDL-C, IDL-C, and VLDL-C are apolipoprotein-B containing particles [71]. Cui et al. have reported that serum non–HDL-C is a stronger predictor of CVD mortality than LDL-C [72]; because it measures all of the potentially atherogenic lipid fractions (LDL-C, IDL-C, VLDL-C, and VLDL remnants).

Wallace et al. demonstrated the effect of high LDL-C on the incidence coronary disease, by demonstrating an association between serum LDL-C levels and single-nucleotide polymorphisms (SNPs) in gene variants of the Proline/serine-rich coiled-coil protein 1 (PSRC1) and Cadherin EGF LAG seven-pass G-type receptor 2 (CELSR2) [52]. Whilst we did not undertake a genetic analysis in our study we did evaluate their family history of CVD. Lloyd-Jones et al. found that the occurrence of parental CVD is an independent predictor of offspring CVD events in middle-aged men and women in a cohort study [57]. Therefore, genetic factors play an important role in the occurrence of dyslipidemia and also CVD events.

Additionally, previous studies showed that unhealthy diets with higher fat and calorie can impose CVD by the effect of dyslipidemia [15], we did not find any relationship between the occurrence of CVD and the quality of diet (HEI).

One strength of our cohort study was that it was performed in a large sample (*n* = 8698). Second, the associations were adjusted for several potential confounding factors, including diet. Also, some limitations of the current study must be considered. Although the great sample size was used in the current study compared to previous studies among the Iranian population, it was not as large as recent investigations that have been undertaken elsewhere [3, 27].

## Conclusion

We found a significant association between total cholesterol and MI risk in this prospective cohort study among middle-aged men of Iran, in the city of Mashhad. Nevertheless, mechanisms that involved in increasing the risk of CVD through lipid profile components are unclear and more studies are needed to determining them.

## Data Availability

Data sharing not applicable to this article as no datasets were generated or analyzed during the current study.

## Abbreviations

AIP: Atherogenic Index of Plasma
ANOVA: Analysis of variance
BMI: Body mass index
CELSR2: Cadherin EGF LAG seven-pass G-type receptor 2
CI: Confidence interval
CT: Computed Tomography
CVD: Cardiovascular disease
DBP: Diastolic blood pressure
DH: Drug history
ECG: Electrocardiogram
ETT: Exercise Tolerance Test
FBG: Fasting blood glucose
FH: Family history
HC: Hip circumference
HDL-C: High-density lipoprotein cholesterol
HR: Hazard ratio
HTN: Hypertension
IDL: Intermediate density lipoproteins
LDL-C: Low-density lipoprotein cholesterol
MAC: Mid-upper arm circumference
MASHAD: Mashhad stroke and heart atherosclerotic disorder
MI: Myocardial infarction
PSRC1: Proline/serine-rich coiled-coil protein 1
SA: Stable angina
SBP: Systolic blood pressure
SNPs: Single-nucleotide polymorphisms
TC: Total cholesterol
TG: Triglycerides
US: Unstable angina
VLDL: Very low-density lipoproteins
WC: Waist circumference
WHO: World Health Organization
WHR: Waist-to-hip ratio
